# Value of Contrast-Enhanced Ultrasound in Guidance of Percutaneous Biopsy in Peripheral Pulmonary Lesions

**DOI:** 10.1155/2015/531507

**Published:** 2015-10-20

**Authors:** Yi Dong, Feng Mao, Wen-Ping Wang, Zhen-Biao Ji, Pei-Li Fan

**Affiliations:** Department of Ultrasound, Zhongshan Hospital, Fudan University, 180 Fenglin Road, Shanghai 200032, China

## Abstract

*Objectives*. To investigate the value of contrast-enhanced ultrasound (CEUS) in guidance of percutaneous biopsy in peripheral pulmonary lesions. *Methods*. This study focused on 53 patients (male: 38, female: 15, and mean age: 55.7 years ± 10.7) with 53 single peripheral pulmonary lesions. Before core needle (16-gauge) percutaneous biopsy, CEUS were performed in all lesions, with injection of 2.4 mL SonoVue (Bracco, Italy). The contrast-enhancement pattern, display rate of internal necrosis (nonenhanced) and active (obviously enhanced) areas, biopsy success rate, and pathological diagnosis rate were recorded. *Results*. All the peripheral pulmonary lesions were proved pathologically as benign lesions (*n* = 7), primary malignancies (*n* = 41), or metastasis (*n* = 5). Forty (86.9%) malignant lesions and 4 (57.1%) benign lesions showed internal necrosis areas on CEUS. The detection rate and average size of internal necrosis areas had been significantly improved compared to conventional ultrasound (*P* < 0.05). After CEUS, core needle percutaneous biopsies were performed successfully in the active areas of all lesions. The sampling success rate and pathological diagnosis rate were 100% and 98.1%. *Conclusions*. CEUS before biopsy provided useful diagnostic information about peripheral pulmonary lesions. By depicting internal necrotic and active areas, it is a promising technique for guaranteeing the accuracy, success, and safety of core needle biopsy.

## 1. Introduction

Percutaneous biopsy guided by ultrasound is a well-established and reliable method for diagnosing peripheral pulmonary lesions, especially when adequate tissue specimens are difficult to be obtained by bronchoscopy [1]. It had similar diagnostic accuracy as biopsy guided by computed tomography (CT) [2]. Under the condition that the peripheral pulmonary lesion had an adequate acoustic widow, ultrasound guidance owned its distinguished advantages such as real-time monitoring of the needle tip, no radiation exposure, low cost, bedside capabilities, and time savings [3]. During the ultrasound guided biopsy, we can choose the safe biopsy route avoiding puncturing lung or large vessels, and the needle can be advanced with safe depth [4].

However, according to literature, false-negative results were occasionally encountered in 15% of patients [5], and the main cause for false-negative result was inadequate specimens and extensive lesion necrosis. As conventional ultrasound could not effectively distinguish necrotic areas in 9%–26% of patients when the necrotic component is very large [4, 6], various methods had been applied to improve biopsy accuracy and to obtain adequate representative specimens, including using new biopsy needles, performing repeated biopsies, or making a cytopathologist present during biopsy [7]. At the same time, the occurrence rate of complications may be increased with applications of those methods.

In recent years, contrast-enhanced ultrasound (CEUS) is widely used in the diagnostic imaging of abdominal organs, especially in focal liver lesions [8, 9]. The lung has dual blood supply (pulmonary arteries and systemic bronchial arteries); theoretically it was considered to be suitable for CEUS evaluation of arterial vascularity [1, 10]. Previously, several literatures suggested CEUS were useful in differentiation diagnosis of peripheral pulmonary lesions [11, 12].

The second generation ultrasound microbubbles are blood-pool agents; contrast-enhancement represented a perfect tool for assessing regional blood perfusion in real time [13]. We hypothesized that CEUS could differentiate internal necrosis or active tissue of peripheral pulmonary lesions before percutaneous core needle biopsy and improve the diagnostic efficacy. Therefore, the purpose of our study was to investigate the value of CEUS in real-time guidance of percutaneous biopsy in peripheral pulmonary lesions.

## 2. Materials and Methods

The study was approved by Ethics Committee of our hospital and informed consent was obtained from each patient. Between September 2004 and October 2014, the patient population consisted of 73 patients (male, 54; female, 19; mean age, 51.0 years ± 14.2) consecutively admitted to our hospital. Those patients had newly discovered single focal lesions located in the peripheral regions of the lung.

The inclusion criteria were the following: (1) patients referred to the Department of Ultrasound in our institution for ultrasound guided percutaneous biopsy of pulmonary peripheral lesions and (2) pulmonary peripheral lesions that were detected on contrast-enhanced CT within one month and could have been visualized at conventional gray-scale ultrasound.

Exclusion criteria were (1) patients who had recent myocardial infarction or pulmonary hypertension or adult respiratory distress syndrome, (2) patients who were unable to undergo imaging guiding biopsy with poor cooperation, (3) contraindications for application of CEUS, such as impaired cardiopulmonary function or known allergic reactions, and (4) patients who had bleeding tendency (prothrombin activity < 40%, international normalized ratio > 1.7, and platelet count < 40,000/mL).

### 2.1. Ultrasound Methods

Before biopsy, conventional ultrasound scanning and CEUS were performed with C5-2 broadband curved transducer of HD15 units (Philips, Bothell, WA, USA) in all lesions. Patients were examined in the lateral or the supine position with their arms lifted above the heads or on the contralateral shoulders to expand the intercostal spaces and access the subscapular region. Chest contrast-enhanced CT was performed in all patients before our study. First, with the reference to the results of chest CT, conventional gray-scale ultrasound was performed to detect the lesion, to observe the lesion's location, size, internal echo, and color flow distribution. We observed from the intercostal, subcostal, or parasternal approach, and choose the optimal acoustic window for CEUS examination.

Then, CEUS were performed with a low mechanical index (MI) 0.05~0.12 with injection of 2.4 mL SonoVue (Bracco S.p.A., Milan, Italy) into an antecubital vein via a 20-gauge cannula followed by 5 mL saline flush. These lesions were continuously examined for at least 180 seconds taking care to include a portion of normal surrounding parenchyma in the same US scan in order to examine the enhancement of both the lesion and normal lung in real time. If normal lung parenchyma could not be included in the same imaging scan, or the lesion was located at the basis of the lung, the surrounding chest wall or the liver (lesion in right lung) or spleen (lesion in left lung) was examined contemporaneously to the lesion [7].

All ultrasound and CEUS examinations were performed by one physician with 10 years of experience in CEUS examination. The clips were recorded, stored, and independently reviewed by the two readers. The enhancement patterns (homogeneous or inhomogeneous) and active (obviously enhanced) and necrosis (nonenhanced) areas were recorded. Active areas were most obviously enhanced regions of lesions and necrosis areas were anechoic regions without enhancement. If there existed any discordance in the evaluation of the CEUS enhancement between the two readers, the clip was reviewed and discussed with a third reader to reach the final decision by consensus.

### 2.2. Ultrasound Guided Biopsy

Immediately after the CEUS, the optimal biopsy routes and sampling sites were selected, including the active area and avoiding the internal necrosis area or large vessels. Then biopsies were performed under real-time guidance of ultrasound. After the skin was sterilized and local anesthetic (2% lidocaine) was injected, biopsies were performed with 16-gauge core tissue biopsy needle and Bard Magnum biopsy instrument (Bard Peripheral Vascular Inc., USA). Patients were instructed to suspend respiration and the needle was advanced into the target active area under real-time guidance. For each patient, two 15 mm long core specimens were obtained. Before being immersed in 10% formalin, the biopsy specimen was placed on a small piece of filter paper and was checked by the operator to evaluate whether the specimen was adequate or the biopsy was successful. The specimens were later sent for histopathological examination. After the biopsy procedure, patients were closely monitored for 3 to 4 hours.

### 2.3. Statistical Analysis

Data were expressed as mean ± standard deviation. All statistical analyses were performed with SPSS 15.0 software package (SPSS, Chicago, IL, USA). The differences between conventional ultrasound and CEUS were evaluated with paired *t*-test. A difference was considered statistically significant with *P* < 0.05. Kappa statistics were calculated to assess interobserver agreement.

## 3. Results

### 3.1. Peripheral Pulmonary Lesions

**Figure 1:**
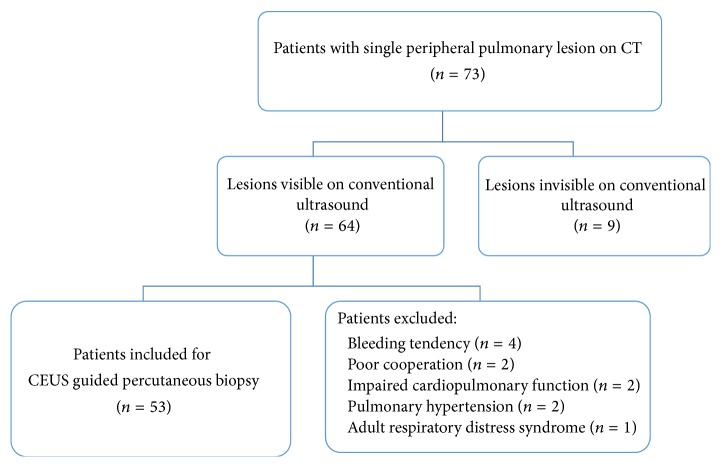
Flow diagram of the patient selection for CEUS guided percutaneous biopsy in peripheral pulmonary lesions.

The final patient population consisted of 53 patients (male, 38; female, 15; mean age, 55.7 years ± 10.7), with newly discovered 53 single focal lesions located in the peripheral regions of the lung (Figure 1).

Twenty-three pulmonary lesions were located in the left lung (12 in upper lobe and 11 in inferior lobe). Thirty lesions were located in the right lung (8 in the upper lobe, 12 in the middle lobe, and 10 in the inferior lobe). Biopsy histopathological diagnosis included 46 malignant and 7 benign lesions. Among 46 malignant lesions, 18 (39.1%, 18/46) were adenocarcinomas, 11 (23.9%, 11/46) were squamous-cell carcinomas, 12 (26.1%, 12/46) were small-cell carcinomas, and 5 (10.8%, 5/46) were metastatic malignant lesions. The other 7 lesions were benign, including 2 lipomas, 2 tuberculous granulomas, 2 abscesses, and one chondroid hamartoma.

### 3.2. Conventional Ultrasound

The mean maximum diameter of peripheral pulmonary lesions was 30.4 ± 7.7 mm (mean ± SD). All lesions were hypoechoic on grey-scale ultrasound, including 11 homogeneous ones and 42 heterogeneous ones. The arterial flow was detected by color Doppler in 18 lesions.

### 3.3. CEUS

CEUS examinations were successfully performed in all 53 patients and no adverse reaction of SonoVue was observed.

**Figure 2:**
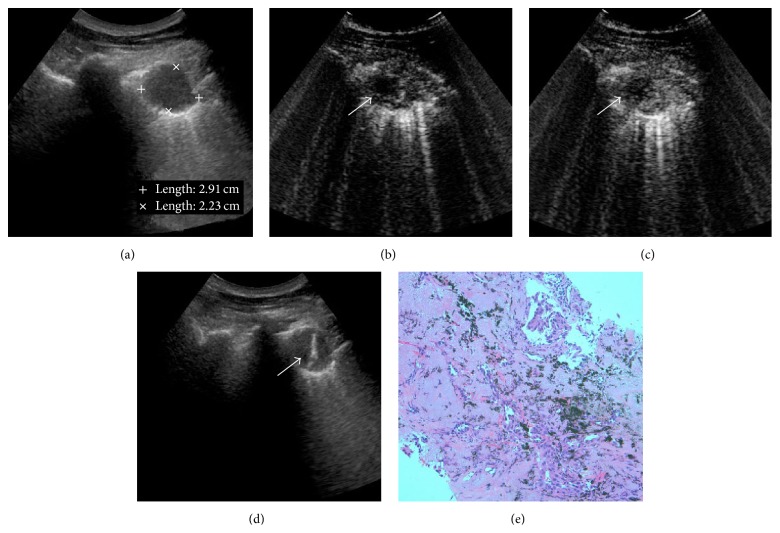
CEUS guided percutaneous biopsy of lung adenocarcinomas. (a) Gray-scale ultrasound detected a hypoechoic and homogeneous lesion in the upper lobe of right lung; no necrotic area was shown with conventional ultrasound. (b) After injection of SonoVue, the lesion showed rapid and heterogeneous enhancement. The enhancement began approximately at 16 sec; a small anechoic necrotic area was detected in the lesion (arrow). (c) Enhancement of lesion remained for approximately 115 sec, with unenhanced necrotic area inside it (arrow). (d) After CEUS, 16-gauge core needle percutaneous biopsy was performed successfully in the active areas of the lesion. The necrotic area was avoided during the real-time ultrasound guidance. (e) Biopsy histopathological diagnosis revealed it was a lesion of lung adenocarcinomas.

**Figure 3:**
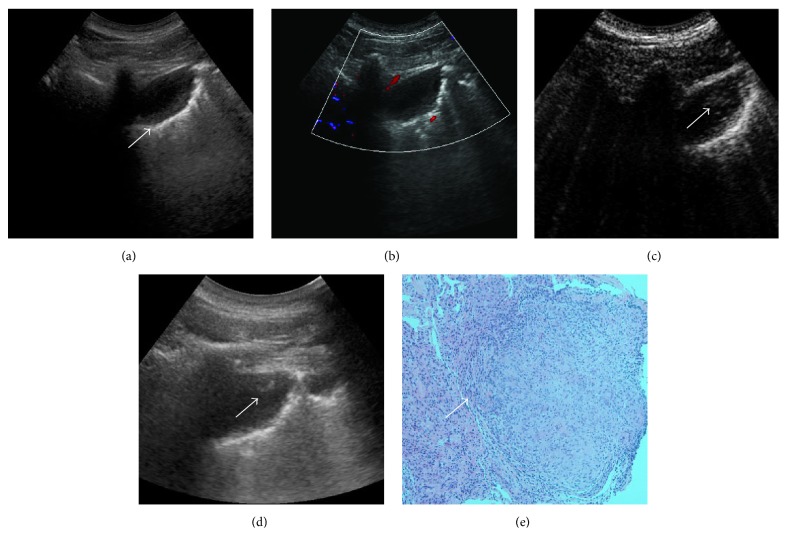
CEUS guided percutaneous biopsy of lung tuberculosis. (a) Gray-scale ultrasound detected a hypoechoic and homogeneous lesion in the upper lobe of left lung. (b) CDFI detected no color flow inside the lesion. (c) After injection of SonoVue, the enhancement began approximately at the peripheral region from 11 sec (arrow). (d) After CEUS, 16-gauge core needle percutaneous biopsies were performed under the real-time guidance of ultrasound in the active area (the lesion's peripheral region). (e) Biopsy histopathological diagnosis revealed it was a lesion of lung tuberculosis, with neutrophils, lymphocytes, and mononuclear cell infiltration.

**Figure 4:**
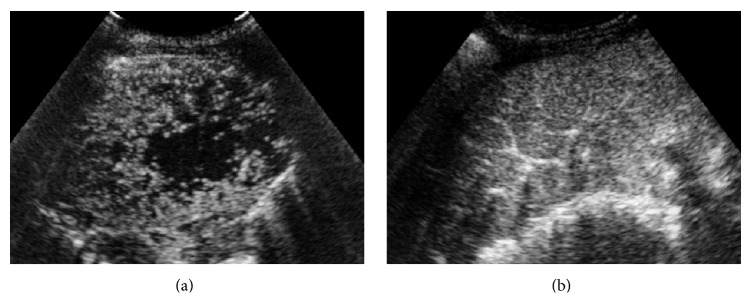
Real-time CEUS showed different vascular distribution in peripheral pulmonary lesions. (a) Disorderly blood vessels in malignant lesion after injection of SonoVue. Biopsy histopathological diagnosis revealed it was a lesion of squamous-cell lung carcinoma. (b) Regular blood vessel and branching vascular distribution enhancement in a lesion of tuberculous granuloma.

After SonoVue administration, rapid and heterogeneous enhancement was observed in 93.4% (43/46) malignant and 57.1% (4/7) benign lesions (*P* < 0.05) (Figures 2 and 3). Real-time CEUS showed relatively regular blood vessel or branching vascular distribution in 4 benign lesions and disorderly blood vessels in 29 malignant lesions (Figure 4).

**Figure 5:**
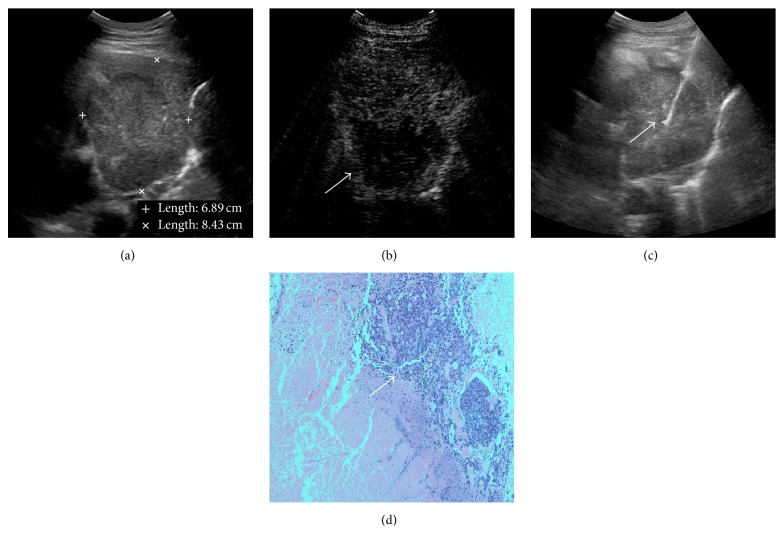
CEUS guided percutaneous biopsy of peripheral pulmonary lesion with large area of necrosis. (a) Gray-scale ultrasound detected a hypoechoic and heterogeneous lesion in the middle lobe of right lung; no necrotic area was shown with conventional ultrasound. (b) After injection of SonoVue, the lesion showed rapid and heterogeneous enhancement in the peripheral region. A large necrotic area without enhancement was detected in the lesion (arrow). (c) After CEUS, 16-gauge core needle percutaneous biopsies were performed under the real-time guidance of ultrasound in the active area (arrow). (d) Histopathological diagnosis revealed it was a lesion of small-cell lung cancer.

Before CEUS, twelve (25.5%, 12/47) malignant and 2 (28.5%, 2/7) benign lesions showed anechoic necrotic areas. After administration of SonoVue, forty (85.1%, 40/47) malignant and 4 (57.1%, 4/7) benign lesions showed nonenhanced necrotic areas (Figure 5). The detection rate of internal necrosis areas had been significantly improved (*P* < 0.05). The average diameter of necrotic areas after CEUS was 14.9 ± 8.4 mm (mean ± SD), ranging from 6 mm to 23 mm. Compared with the mean size before CEUS (7.9 ± 5.2 mm), the necrotic areas after CEUS had been more accurately defined (*P* < 0.05). Necrosis was most common in squamous-cell lung carcinomas (81.8%, 9/11), the adenocarcinomas (72.2%, 13/18), and tuberculous granulomas (40%, 2/5).

In all cases good interreader agreement (*κ* = 0.790) was achieved after two observers reviewed and discussed the CEUS clips.

### 3.4. Biopsy

After CEUS, the percutaneous biopsies with 16-gauge core needles were performed successfully in the active areas of all lesions. With the CEUS guidance, we arranged the safe biopsy routes, including the obviously enhanced active areas of the lesions and avoiding large vessels. In the lung lesions with unenhanced necrotic areas, biopsies were performed by CEUS guidance to avoid the necrosis area.

The biopsy successful rate with CEUS guidance was 100% (53/53). In 98.1% (52/53) of cases, specimens biopsied under CEUS guidance were adequate for pathologic diagnosis. None of the patients had adverse reactions or biopsy complications.

## 4. Discussions

Previously, without CEUS, conventional ultrasound was routinely used for selection of an optimal biopsy needle path [14]. However, it depended on the examiner and was not sensitive in detecting low velocity flow signals in small vessels. Nowadays, CEUS in the characterization of peripheral pulmonary lesions has been investigated in a few preliminary studies [15, 16]. Those pilot investigations were mainly descriptive and showed that, with reference to the time of enhancement and enhancement pattern, CEUS was helpful for differentiating the peripheral pulmonary tumor from pleurisy, embolism, pneumonia, or atelectasis of different causes [10–12, 14, 17].

The lung is characterized by dual blood supply, which provides theoretical base for CEUS to evaluate the blood perfusion of peripheral pulmonary tumors. The pulmonary artery blood supply is responsible for gas exchange, and tumor angiogenesis usually rises from the bronchial artery blood supply [10, 14]. Consistent with the results of our study, delayed enhancements in malignant lesions were likely determined by widespread vasoconstrictions caused by hypoxia [17–19]. And the faster arterial enhancements in benign lung lesions were due to the dual blood supply [14]. Moreover, a relatively regular or branching vascular distribution was more often observed in benign or nonneoplastic lung lesions, which was only supplied by the pulmonary arteries [18]. We observed regular blood vessel or branching vascular distribution in the 4 benign ones and disorderly blood vessels in 29 malignant ones during the CEUS enhancement. These characteristics may provide a general indicator of the probability of malignancy or benign peripheral pulmonary lesions before biopsy, which may avoid unnecessary biopsy in certain benign cases.

Hemoptysis and pneumothorax are the most frequent complications of core needle biopsy of peripheral lung lesions. Compared to real-time observation of CEUS, contrast-enhanced CT can only evaluate the vascularity of lung lesions at a certain point during the arterial phase [20]. CEUS could effectively and dynamically detect peripheral lung lesions' vascularization. Additionally, during the arterial phase of enhancement, real-time CEUS may be convenient and sensitive to detect large vessels inside the lesions, which can be avoided during the following biopsy. In this way, severe complications such as bleeding and pneumothorax can be avoided by choosing the safe biopsy route and advancing the needle with safe depth during the ultrasound guided biopsy.

In the literature, the diagnostic accuracy of peripheral pulmonary lesions biopsy performed under conventional gray-scale ultrasound guidance was reported from 91% to 96% [21, 22]. A small percentage of specimens were inadequate for diagnosis because of their inner necrotic areas [5]. The presence of a higher percentage of tumor necrosis also accounted for the lower diagnostic accuracy rate for tumors larger than 5 cm in diameter [22]. Pathologically, the low blood supply of bronchial artery during the progress of lung tumor related to high incidence of internal necrotic rate. In our present study, CEUS could delineate lesion necrosis as nonenhanced regions and active areas as hyperenhanced regions with high sensitivity and specificity, which can hardly be detected by conventional ultrasound. We avoided the necrotic areas and biopsied hyperenhanced active areas, where sampling specimens were thought to be more reliable for histologic diagnosis [23]. The biopsy successful rate with CEUS guidance was 100% (53/53) in our study. Additionally, 98.1% of patients had sufficient specimens for histologic diagnosis. Compared with the results of literatures, CEUS significantly improved the diagnostic accuracy of initial biopsy of peripheral lung lesions and avoided multiple biopsies. In addition, in tumors with pulmonary infiltrates, CEUS could easily differentiate tumors from the homogeneous enhanced consolidated lung tissues and avoid unnecessary biopsy of lung consolidations or infiltration lesions.

Our study has several limitations. First, CEUS are strictly operator-dependent techniques. Second, because of the limited number of patients enrolled in our study, benign neoplastic lesions were only 13.2% (7/53).

## 5. Conclusions

In conclusion, real-time CEUS can easily depict internal necrotic and active areas. The prebiopsy CEUS is a promising technique for reducing the risk of inadequate tissue sampling and guaranteeing accuracy and success of core needle biopsy.
